# Supplementary material for: Evaporation-driven transport-control of small molecules along nanoslits
Source: Nat Commun. 2021 Feb 26;12:1336. doi: 10.1038/s41467-021-21584-8 (PMC7910579; doi:10.1038/s41467-021-21584-8)
Supplement: Supplementary file 1 — Supplementary Information [file 41467_2021_21584_MOESM1_ESM.pdf]

***Supplementary Information*:**

Evaporation-driven transport-control of small molecules along nanoslits

Sangjin Seo[†], *Dogyeong Ha*[†], *and Taesung Kim*[*]

Department of Mechanical Engineering, Ulsan National Institute of Science and Technology (UNIST), 50 UNIST-gil, Ulsan 44919, Republic of Korea

[†]These authors contributed equally to this work.

CORRESPONDENCE:

Taesung Kim
Department of Mechanical Engineering
Ulsan National Institute of Science and Technology (UNIST)
50 UNIST-gil, Ulsan 44919, Republic of Korea
E-mail: tskim@unist.ac.kr
Tel: +82-52-217-2313
Fax: +82-52-217-2449

Supplementary Notes

Supplementary Note 1: Dynamics of solute transport along the nanoslit

In the nanoslit, the mass transport of the solute is governed by the advection-diffusion equation:

$$\frac{\partial c}{\partial t} = \nabla \cdot (D \nabla c) - \nabla \cdot (\boldsymbol{v} c) , \tag{1}$$

where $c = c(x,t)$ is the solute concentration, D is the diffusion coefficient of the solute, and $\boldsymbol{v} = \boldsymbol{v}(x,t)$ is the advective flow velocity. Thus, the first term on the right-hand side of Supplementary Equation (1) represents the diffusive transport of the solute while the second term represents the advective transport of the solute. The averaged flow velocity driven by solvent evaporation through the nanoslit walls with a constant evaporation flux was approximated for a 1D model as follows:[1]

$$\boldsymbol{v} = v_{x,\text{avg}}(x) = -\frac{Jx}{\rho h} , \tag{2}$$

where J is the evaporation flux of the solvent through the nanoslit walls, ρ is the solvent density, and h is the nanoslit height. Therefore, the governing equation was simplified as follows for a low Péclet number (i.e., $\text{Pe}_h = \frac{v_{x,\text{avg}}(x=L)h}{D} = \frac{JL}{\rho D} < 9 \times 10^{-3}$):

$$\frac{\partial c}{\partial t} = \frac{\partial}{\partial x}(D\frac{\partial c}{\partial x}) + \frac{\partial}{\partial x}(\frac{Jx}{\rho h} c) . \tag{3}$$

In the dimensionless form, this becomes

$$\frac{\partial \tilde{c}}{\partial \tilde{\tau}} = \frac{t_o D}{L^2}\frac{\partial^2 \tilde{c}}{\partial \tilde{x}^2} + \frac{t_o J}{\rho h}\tilde{x}\frac{\partial \tilde{c}}{\partial \tilde{x}} + \frac{t_o J}{\rho h}\tilde{c} ; \tag{4}$$

$$\tilde{c}(\tilde{x}, \tilde{\tau} = 0) = 1 \quad -1 \le \tilde{x} \le 1 ; \qquad \text{(Initial condition)}$$

$$\begin{cases} \tilde{c}(\tilde{x} = -1, \tilde{\tau}) = 1 \\ \tilde{c}(\tilde{x} = 1, \tilde{\tau}) = 0 \end{cases} ; \qquad \text{(Boundary conditions)}$$

where $\tilde{c}(\tilde{x},\tilde{\tau}) = \frac{c(\tilde{x},\tilde{\tau})}{c_o}$ is the dimensionless solute concentration, $\tilde{\tau} = \frac{t}{t_o}$ is the dimensionless time, $\tilde{x} = \frac{x}{L}$ is the dimensionless distance, c_o is the solute concentration in the reservoir, t_o is the dehydration time, and the nanoslit length is defined as 2L.

The large dimensional difference between the nanoslit at nanoscales and the test chamber at microscales makes the simulation of the entire device challenging, in terms of time, cost, and accuracy. In addition, for the parametric study, 1D approximation was conducted by focusing on the nanoslit of interest. In this context, we assumed that the concentration of the solution at the drain-channel-sided end of the nanoslit ($x = L$) is zero. This assumption represents a conditional set because the molecules concentrated at the nanoslit originate only from the source channel and not from the drain channel. For our micro/nanofluidic system, the test chamber has a significantly larger cross-sectional area orthogonal to the diffusion direction than that of the nanoslit. Because of this substantial difference in the cross-sectional areas, the concentration gradient drops mostly within the nanoslit to satisfy the continuity of the solute transport. Therefore, even if the concentration in the test chamber is not exactly zero, its value is significantly low in comparison with that in the nanoslit. By separating the nanoslit and the test chamber under this assumption, the concentration of the test chamber represents the mass transport rate from the nanoslit to the test chamber rather than the concentration at the end of the nanoslit ($x = L$). From this point of view, the nanoslits of 50, 100, and 200 μm, wherein the solutes are not concentrated, as depicted in Fig. 3c, show good agreement with those depicted in Fig. 2c. In other words, the same linear concentration gradient was observed for the

dimensionless distance and concentration cases. The shorter nanoslit has a steeper concentration gradient, and the diffusion into the test chamber increases according to Fick's law. Therefore, as depicted in Fig. 2b, as the length of the nanoslit decreases, the fluorescence intensity at the steady state increases, and vice versa.

In summary, the fluorescence intensities of the test chamber are a result of the diffusion from the nanoslit to the test chamber; thus, the shorter the nanoslit, the greater the fluorescence intensities at the test chamber. Therefore, we concluded that the results of Fig. 3c and 2b show good agreement. This is why that the test chamber is integrated with the nanoslit to quantify the mass transport rate along the nanoslit.

In particular, we performed numerical simulations on the diffusion along the nanoslit and the test chamber using COMSOL Multiphysics (ver 5.5). The dimensions of the numerical domain are identical with those of the micro-/nanofluidic system described in the manuscript. The dimensionless concentration, $\frac{c}{c_o}$, at the end of the nanoslit (i.e., the junction between the nanoslit and source channel) was set to 1, and the $\frac{c}{c_o}$ at the end of the test chamber was set to 0 (i.e., the junction between the inlet of the test chamber and the drain channel). In the steady-state study, as depicted in Supplementary Fig. 12a, b, the concentration level drops mainly in the nanoslit and the concentration of the test chamber is considerably close to zero, and seems negligible enough, as depicted in Supplementary Fig. 12c. Furthermore, the volume averaged dimensionless concentrations of the test chamber, $\frac{1}{V}\iiint_V \frac{c}{c_o} dV$ were 0.00273, 0.00136,

0.00068, 0.00034 and 0.00017 for 50, 100, 200, 400 and 800 μm, respectively. V is the volume of the test chamber. The results demonstrate a reciprocal relation between the concentration in the test chamber and the nanoslit length, which shows good agreement with the results depicted in Fig. 2b.

Supplementary Note 2: Numerical simulation

We solved Supplementary Equation (4) using a PDEPE solver, the 1D parabolic and elliptic PDE solver provided by MATLAB. 2D and 3D simulations were performed to verify that the 1D simulation was accurate enough to estimate solute transport behavior in the nanoslit. OpenFOAM, an open source computational fluid dynamics (CFD) software, was utilized for the 2D and 3D simulations.[2] Owing to the independent flow velocity with regard to the solute concentration distribution, the flow velocity was solved with icoFoam, an OpenFOAM solver for incompressible, laminar flow using the Navier-Stokes equation (NSE) with the pressure-implicit with splitting of operators (PISO) algorithm.[3] It was assumed that the solvent viscosity was not affected by fluorescence solutes, which had a low solubility (~1 mM). Therefore, the NSE could be de-coupled with solute concentration. The solvent evaporation flux was assumed to be constant and occur parallel to the normal velocity, $\boldsymbol{v}_{\mathrm{wall}} = (J / \rho)\boldsymbol{n}$, where $\boldsymbol{n}$ is the normal vector on the nanoslit wall. Then, the flow velocity was further utilized to calculate the solute transport and distribution using scalarTransportFoam, an OpenFOAM solver for the transport equation of a passive scalar.[4] Finally, we confirmed that the 2D and 3D simulation results demonstrated good agreement with the 1D simulation result, which enabled us to conduct a parametric study with the 1D simulation.

Supplementary Figures

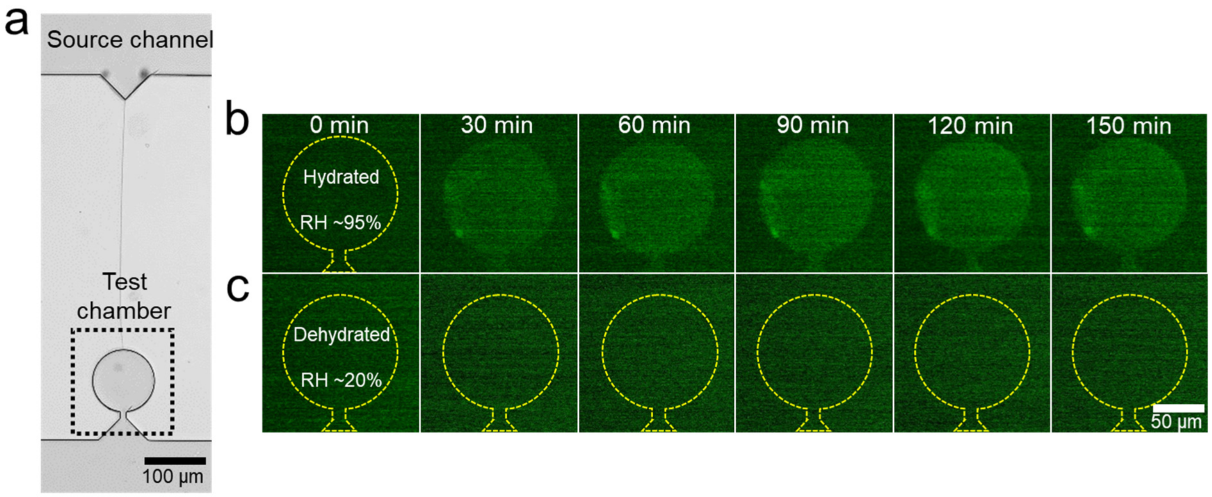

Supplementary Fig. 1 Characterization of fluorescence intensity with test chamber. a Fluorescence intensities (FIs) of the test chamber acquired over time. A global, evaporation-controlled, micro/nanofluidic device (GECMN) with nanoslits of 400 μm is depicted for better presentation of the micro/nanofluidic system, while the experiments were performed using GECMN with nanoslits of 800 μm as depicted in (b), (c) and Fig. 2a. **b** Time-lapse fluorescence images of a fully hydrated polydimethylsiloxane (PDMS) system, which is exposed to a high humidity (relative humidity; RH ~95%) to maintain hydration. The fluorescein isothiocyanate (FITC) molecules are continuously transported along the nanoslit and then arrive at the test chamber. **c** Time-lapse fluorescence images of a fully dehydrated PDMS system, which is exposed to a low humidity (RH ~20%) to maintain dehydration. No FI is detected, confirming that evaporation-driven advective transport of the FITC molecules is stronger than diffusive transport.

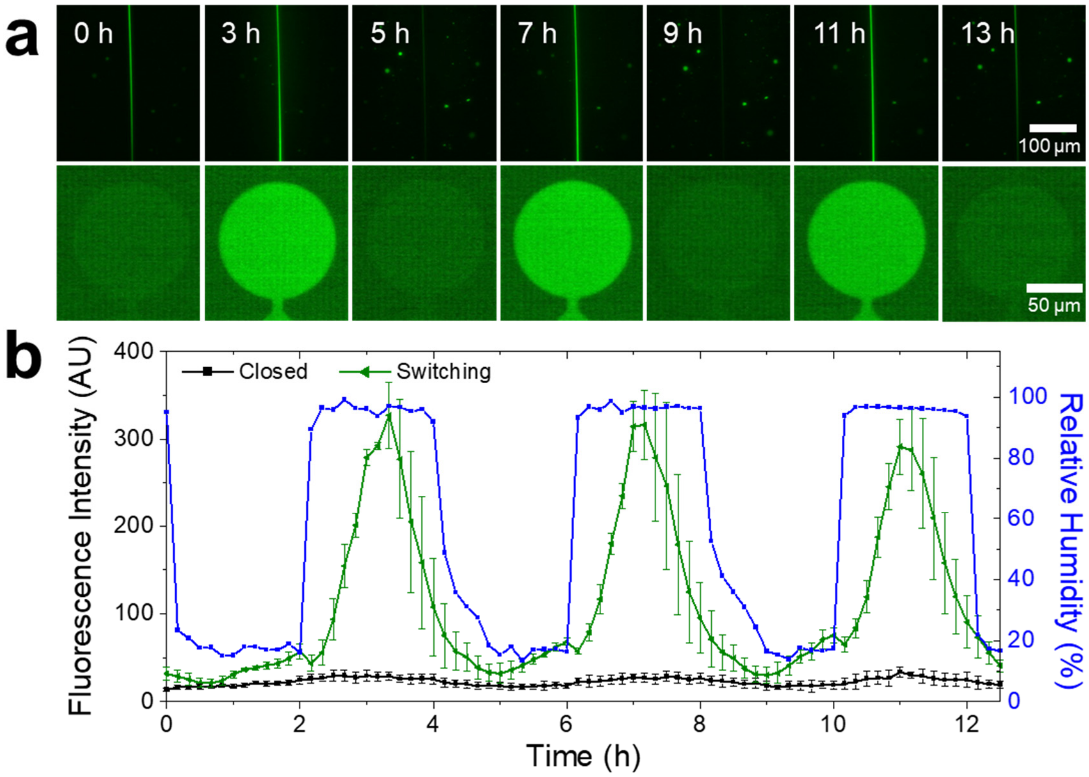

Supplementary Fig. 2 Repeatable switching between dehydration (RH ~20%) and rehydration (RH ~95%) at 2 h intervals, conducted three times to control the mass transport of small molecules. a Time-lapse fluorescence images display the FIs of the 800 μm FITC molecules in the nanoslit and those of the test chamber. **b** Quantification results of the FIs of the test chamber for 12 h. The experiment was performed with three different devices simultaneously to minimize the device-to-device errors. Error bars represents the standard errors on the means (n = 3).

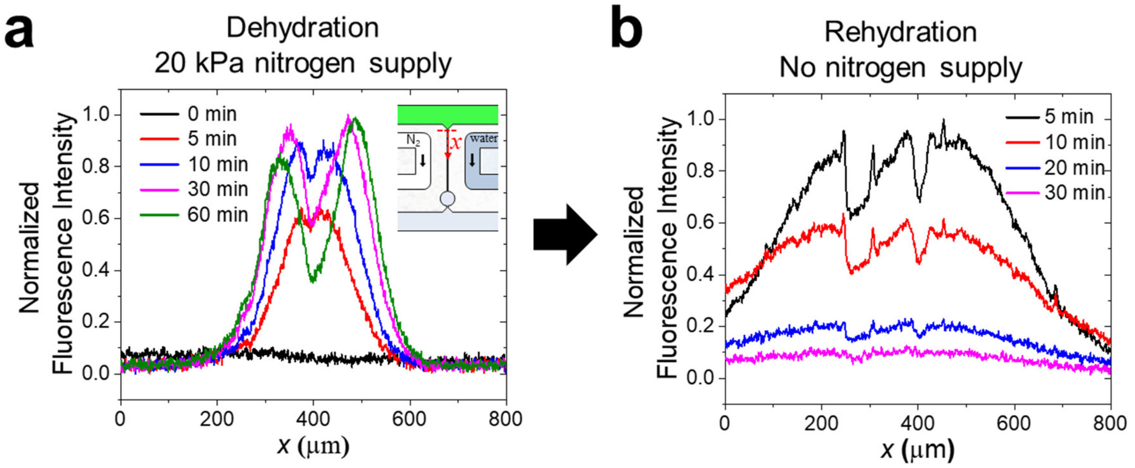

Supplementary Fig. 3 FIs along the nanoslit during dehydration and rehydration. a A high evaporation flux is generated with the dehydrating microchannel by applying 20 kPa of regulator pressure. As the FITC molecules are aggregated/crystalized by an increase in the concentration at the center of the nanoslit, the FIs along the nanoslit display a bimodal graph, resulting in an optical dark region. **b** After rehydration, the enriched FITC molecules at the center of the nanoslit diffuse toward the nanoslit sides such that the FIs at the center of the nanoslit decrease and become linear from the source to the drain channel.

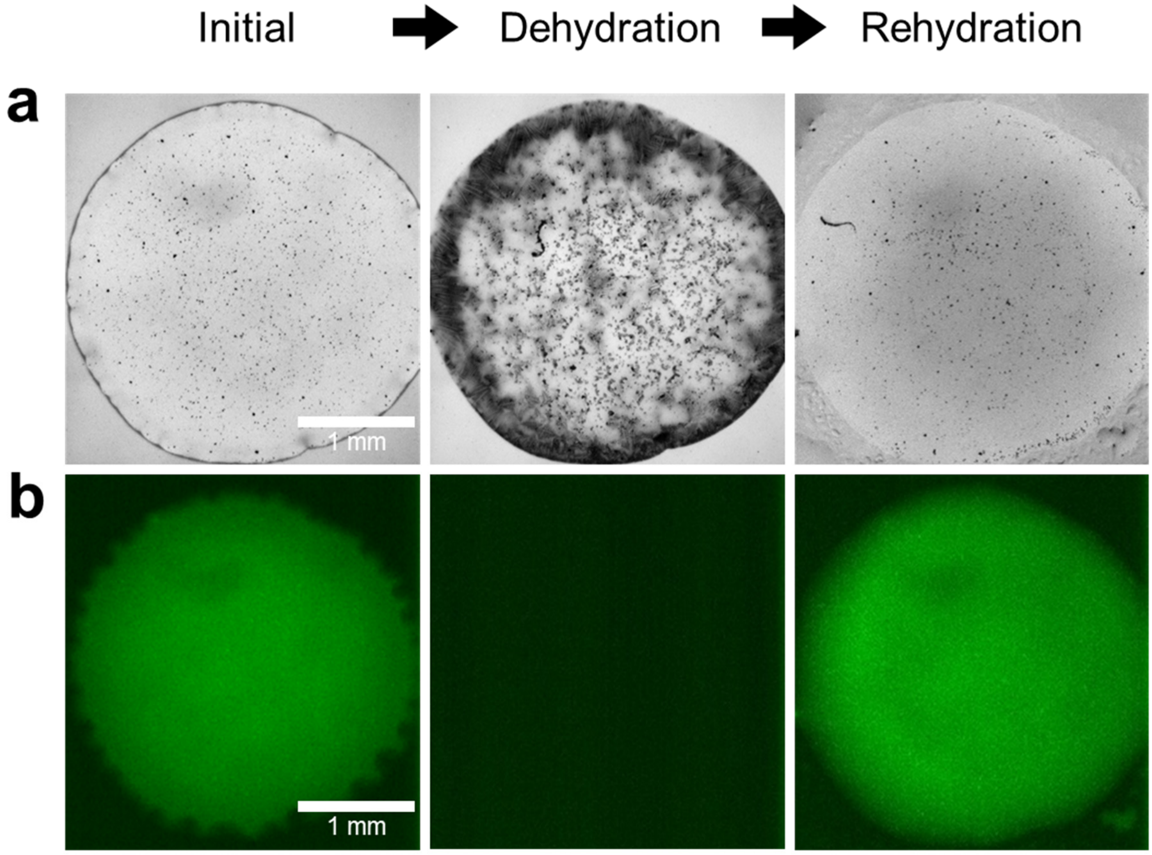

Supplementary Fig. 4 FI experiments using 2.5 μL FITC droplets on a glass substrate indicating that the FI vanishes after aggregation/crystallization with dehydration. a Time-lapse, bright field images of the droplet when the droplet is subjected to dehydration (RH ~20%) for 5 min. The droplet is completely dried out by aggregation/crystallization and then rehydrated at a high humidity (RH ~95%). **b** Fluorescence images of the droplet reveal that the fluorescence signals weakened and disappeared during dehydration but recovered during rehydration.

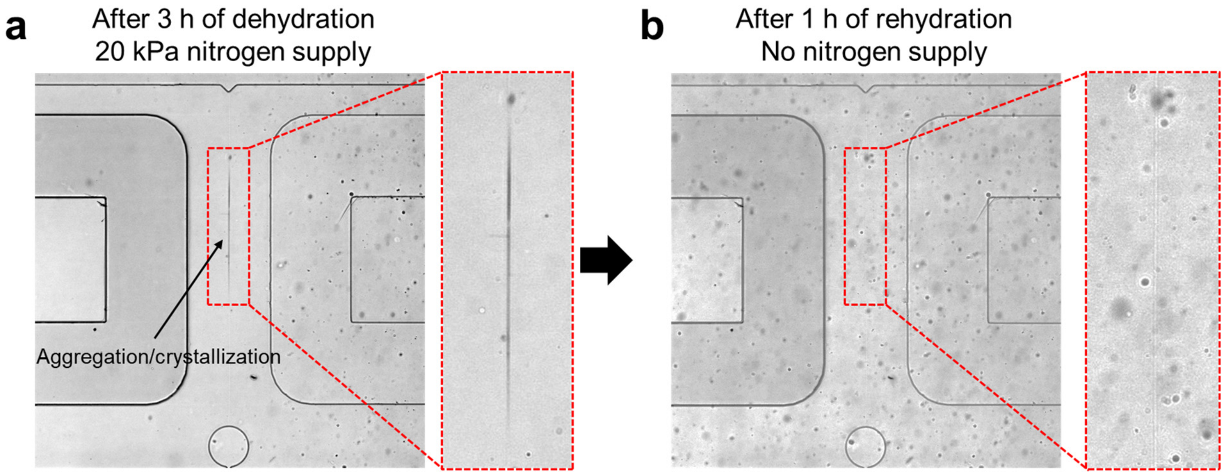

Supplementary Fig. 5 Rehydration after aggregation/crystallization of FITC molecules inside the nanoslit. a After 3 h of dehydration with high concentration of FITC molecules (1 mM). The black arrow indicates the aggregated/crystallized FITC molecules. **b** after rehydration, the concentrated solutes including the aggregated/crystallized FITC molecules, were diffused with no molecules remaining. The rehydration process is shown in Supplementary Movie 3.

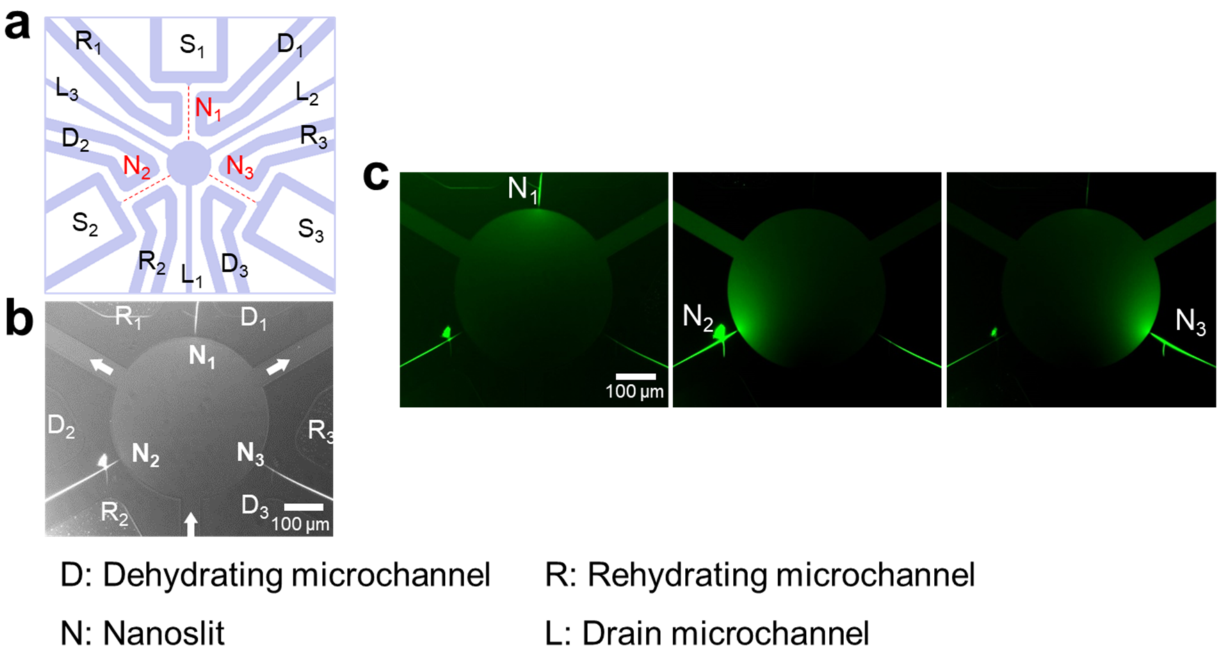

Supplementary Fig. 6 Independent and addressable control of mass transport in a nanoslit array. a A target chamber (circular chamber) is connected to three separate nanoslits (N_1, N_2, and N_3), which are connected to three different source channels (S_1, S_2, and S_3), respectively. Three pairs of dehydrating (D_1, D_2, and D_3) and rehydrating (R_1, R_2, and R_3) microchannels are designed in parallel with each nanoslit. Three drain microchannels from the reservoirs (L_1, L_2, and L_3) are connected to the target chamber at 120° for a symmetric design. **b** Microscopic and fluorescence images illustrate that the target chamber at the center is connected with the three nanoslits and the three microchannels for sample loading as designed. The bottom microchannel (L_1) is used as an inlet for the phosphate-buffered saline (PBS) solution to the target chamber, such that the left-upward (L_3) and right-upward microchannels (L_2) are used as outlets for the PBS solution and FITC molecules from the nanoslits. The FITC molecules diffuse from the target chamber into the nanoslits. **c** The three nanoslits act as the molecule transport gate (i.e., valve) working independently such that the three source molecules are selectively delivered to the target chamber. A sequential gating process is used: N_1 is open, N_2 is open, and then N_3 is open, while the others are closed. Each nanoslit can be individually controlled by the right neighboring dehydration and rehydration microchannels; they are not affected by the other dehydration and rehydration microchannels. Therefore, it is demonstrated that such a micro-/nanofluidic channel network enables mass transport control in an individual, addressable, reversible, and repeatable manner, showing high potential for a variety of micro-/nanofluidic applications on a single chip.

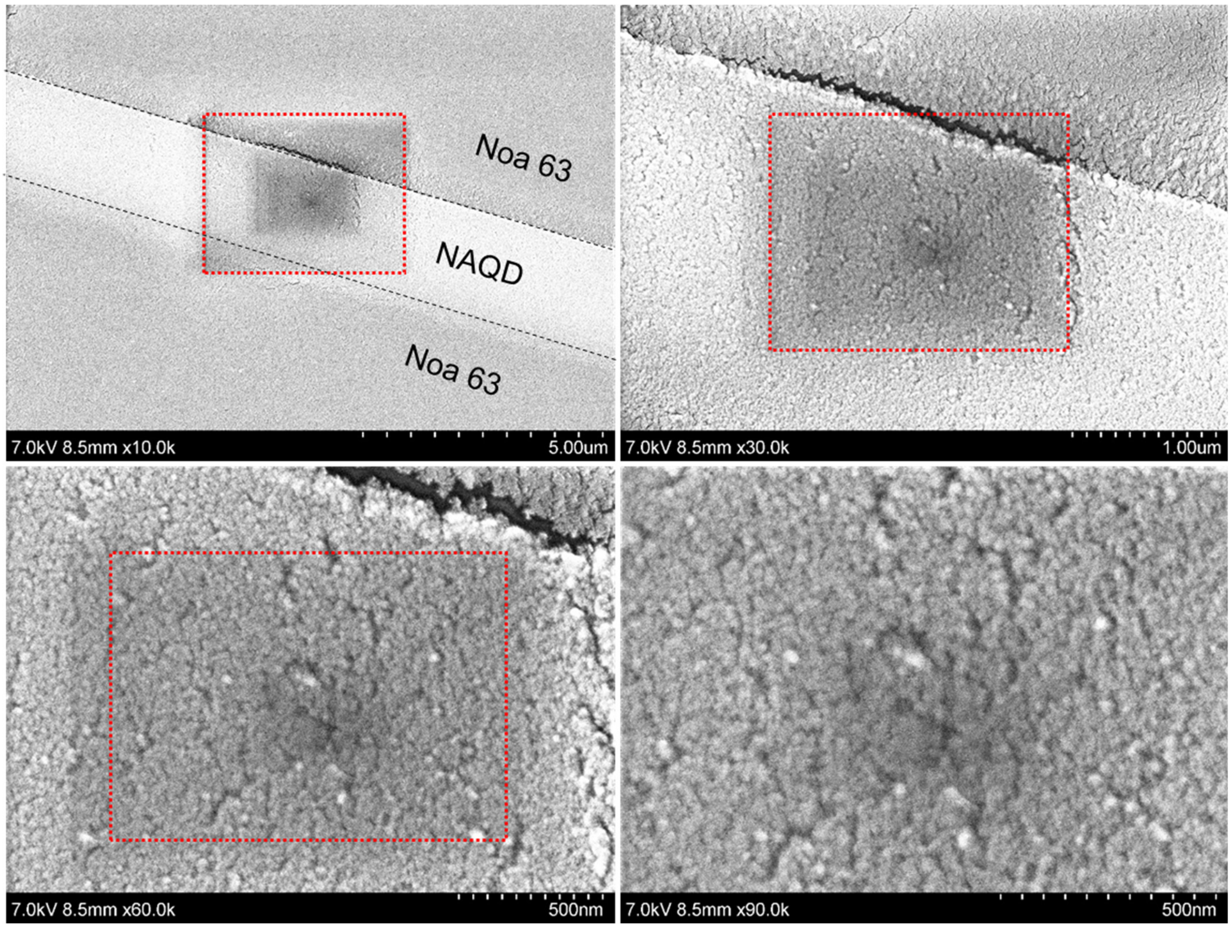

Supplementary Fig. 7 Scanning electron microscopy (SEM) images of the bottom of the nanowire-like assembly of the quantum dots (NAQDs). After lifting off the PDMS device, the NAQD is cleaned by 30 min of sonification while soaked in acetone. Then, the NAQD is transferred from the glass substrate to a UV-curable adhesive, which is immediately cured by a UV lamp before the adhesive permeates into the NAQD structure. Apparently, the SEM images show that the quantum dots (QDs) are close-packed.

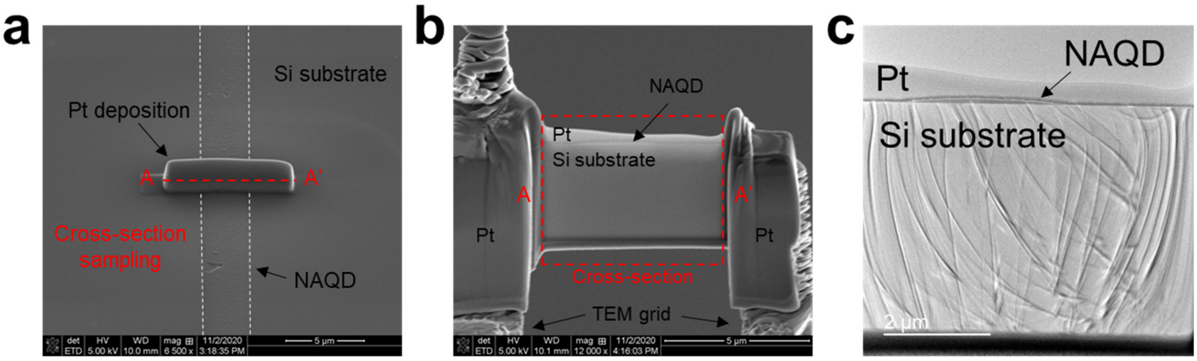

Supplementary Fig. 8 NAQD sample preparation for transmission electron microscopy (TEM) using dual-beam focused ion beam (FIB). The NAQD is generated on the Si substrate. After lifting off the PDMS device, the NAQD is cleaned by sonification for 30 min while soaked in acetone. **a** SEM image of the NAQD deposited with Pt to protect its original structure from FIB milling. The maximal thickness of the NAQD may consist of about 100 QD layers into the depth direction of the Si substrate. **b** SEM image of the cross-section of the NAQD milled by FIB. The cross-section of the sample is delivered to TEM grids and its two ends are fixed by another Pt deposition. **c** TEM image of the cross-section of the NAQD sample.

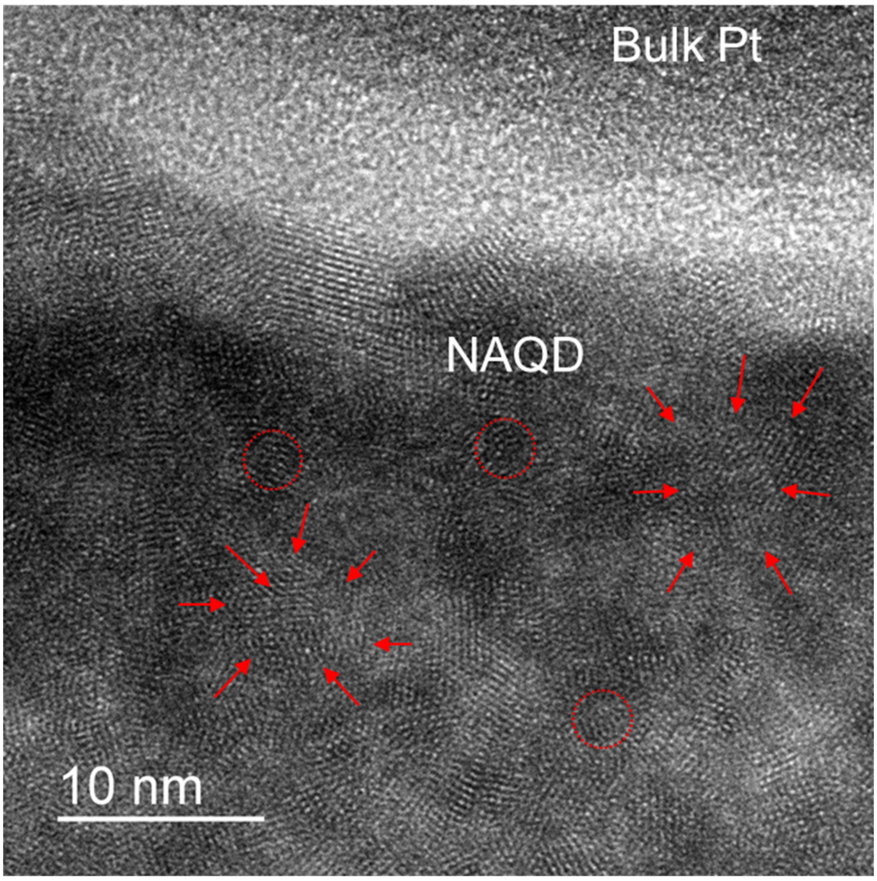

Supplementary Fig. 9 TEM image of the cross-section of the NAQD. It appears that QDs are well assembled in the nanoslit by evaporation-driven advective flow. The lattice spacings of the CdTe crystal structures in the NAQD are distinguishable from those of Pt. The dashed-circles in red indicate the shape of single spherical QDs while the arrows in red do an assembled structure of QDs in part. The NAQD consists of about 50 QD layer into the depth direction of the image that is orthogonal to the cross-section of the NAQD sample as shown in Supplementary Fig. 8b and 8c.

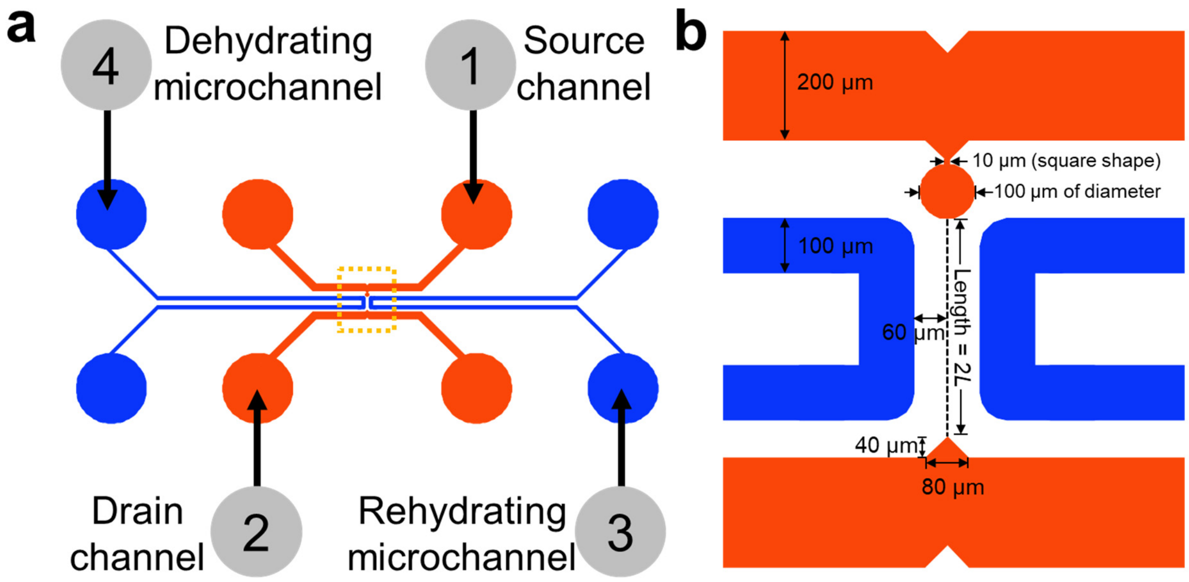

Supplementary Fig. 10 Schematic of local, evaporation-controlled, micro/nanofluidic device (LECMN). The structures of the LECMN and GECMN are identical, except for the absence of the two supporting channels in the GECMN. The height of each microstructure, except for the nanoslit, is 10 µm. (**a**) Schematic of the micro/nanofluidic device with the syringe tubing connections indicated by the numbering in Supplementary Fig. 11. **b** Detailed dimensions of microstructures near the nanoslit (black dashed line).

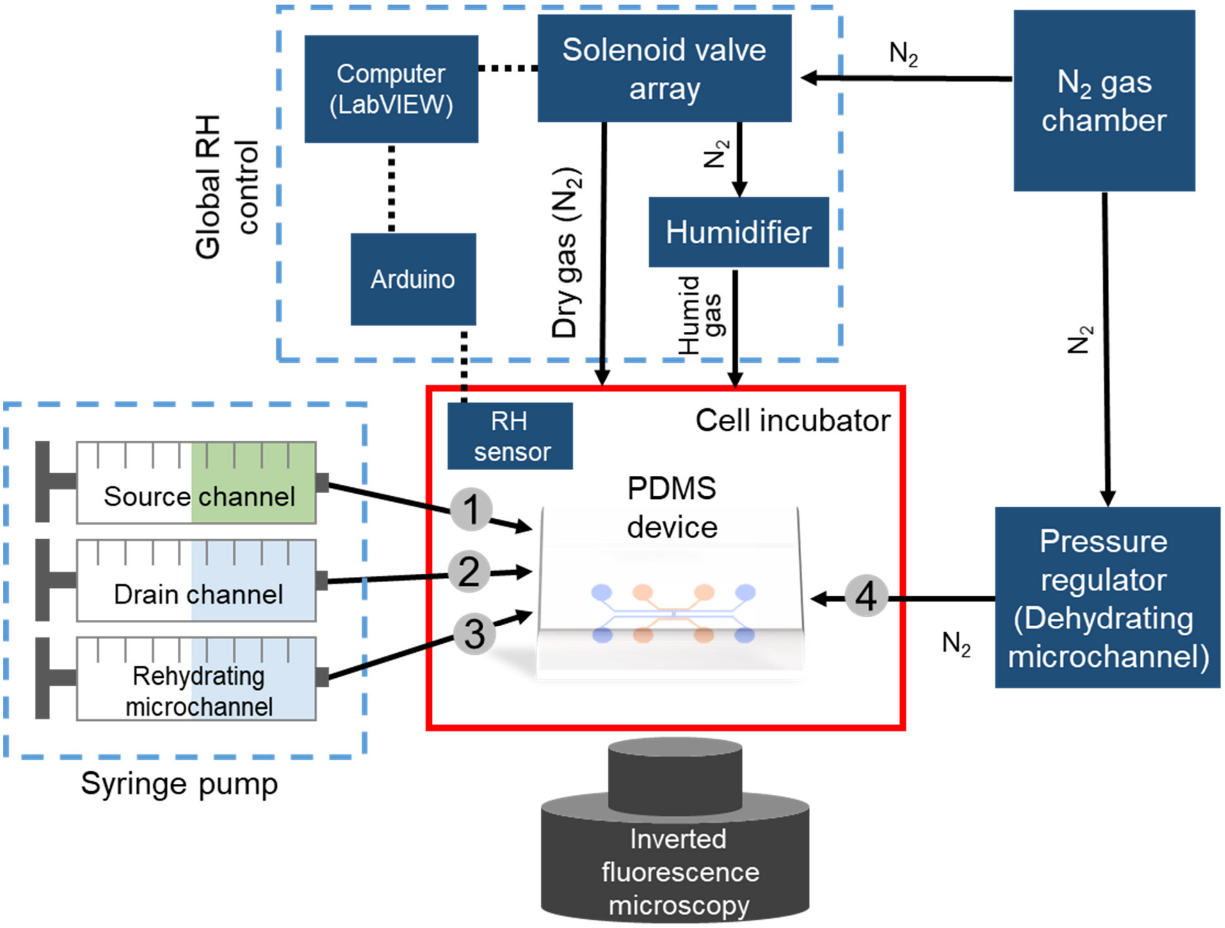

Supplementary Fig. 11 Schematic of experimental setup. Global humidity in the cell incubator is controlled by the Global RH control setup. The RH data measured by the RH sensor are delivered to the computer through a data acquisition system, Arduino. Using the RH data, the computer controls an actuation valve whose inlet is connected to the N2 gas chamber; one of the outlets is directly connected to the cell incubator, and the other outlet is connected to the cell incubator through a humidifier. A syringe pump is used for introducing the solutions into the source, drain, and rehydrating channels at a fixed flow rate. A pressure regulator is connected to the N_2 gas chamber and the device to apply a fixed pressure to the dehydrating microchannel.

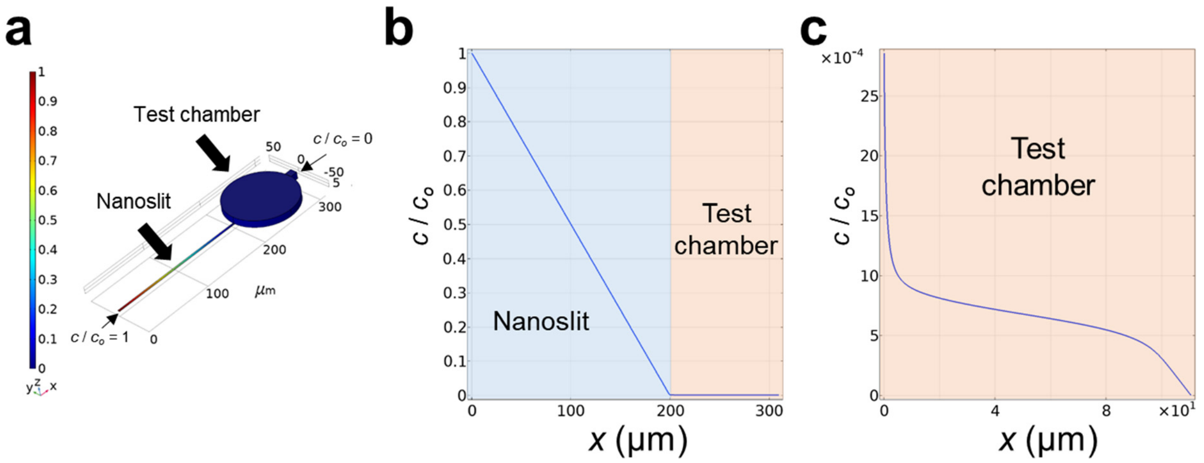

Supplementary Fig. 12 3D numerical simulation results obtained by COMSOL Multiphysics to validate the boundary condition in 1D simulation when only diffusion is considered. The dimensions of the numerical domain are identical to those of the micro-/nanofluidic channel structure described in the manuscript. **a** Concentration distribution of the entire numerical domain when only diffusion is considered. **b** Concentration distribution along the nanoslit from the source channel (x = 0 μm in Supplementary Fig. 12a) to the inlet of the test chamber (x = 310 μm in Supplementary Fig. 12a); all calculations in the measurement are conducted at y = 0 μm and z = 0.1 μm as depicted in Supplementary Fig. 12a, 100 nm above the substrate. The concentration drops significantly inside the nanoslit. **c** The concentration distribution of the test chamber is rescaled from that in (b) to focus on the test chamber. The level of the concentration is considerably low in comparison with that in the nanoslit.

Supplementary References

[1] Randall, G. C. & Doyle P. S. Permeation-driven flow in poly(dimethylsiloxane) microfluidic devices. *Proc Natl Acad Sci U S A* **102**, 10813-10818 (2005).

[2] Weller, H. G., Tabor G., Jasak H. & Fureby C. A tensorial approach to computational continuum mechanics using object-oriented techniques. *Computers in Physics* **12**, (1998).

[3] Issa, R. I. Solution of the implicitly discretised fluid flow equations by operator-splitting. *Journal of Computational Physics* **62**, 40-65 (1986).

[4] Nieves-Remacha, M. J., Kulkarni A. A. & Jensen K. F. Openfoam computational fluid dynamic simulations of single-phase flows in an advanced-flow reactor. *Industrial & Engineering Chemistry Research* **54**, 7543-7553 (2015).

- END -
