# Supplementary material for: Evaporation-driven transport-control of small molecules along nanoslits
Source: Nat Commun. 2021 Feb 26;12:1336. doi: 10.1038/s41467-021-21584-8 (PMC7910579; doi:10.1038/s41467-021-21584-8)
Supplement: Supplementary file 3 — Description of Additional Supplementary Files [file 41467_2021_21584_MOESM3_ESM.docx]

**Description of Additional Supplementary Files**

File name: Supplementary Movie 1

Description: Dehydration and rehydration of the nanoslit with ambient humidity control.

File name: Supplementary Movie 2

Description: FITC solution droplet during (left) dehydration and (right) rehydration.

File name: Supplementary Movie 3

Description: Rehydration of the nanoslit, wherein FITC molecules are aggregated/crystallized inside the nanoslit.

File name: Supplementary Movie 4

Description: Programmable transport control of molecules by repeating rehydration and dehydration with various dehydration times over a long period, as depicted in Fig. 4c.

File name: Supplementary Movie 5

Description: Growth of a nanowire-like QD assembly during dehydration with 20 kPa of applied pressure as a (top) movie and (bottom) fluorescence signal along the nanoslit.

File name: Supplementary Movie 6

Description: Molecular filtering of the solution mixture of two small molecules, acriflavine (blue) and sulforhodamine B sodium (red).

File name: Supplementary Movie 7

Description: Increase in the concentration of acriflavine in the nanoslit with a high solvent evaporation flux.
